# Identification of Diagnostic Exosomal LncRNA-miRNA-mRNA Biomarkers in Colorectal Cancer Based on the ceRNA Network

**DOI:** 10.3389/pore.2022.1610493

**Published:** 2022-09-16

**Authors:** Yajing Zhao, Xingguo Song, Xianrang Song, Li Xie

**Affiliations:** Department of Clinical Laboratory, Shandong Cancer Hospital and Institute, Shandong First Medical University and Shandong Academy of Medical Sciences, Jinan, China

**Keywords:** colorectal cancer, lncRNA, miRNA, exosomes, diagnostic biomarker, mRNA

## Abstract

**Background:** Colorectal cancer (CRC) is currently the fourth most common cancer worldwide. The roles of exosomal competing endogenous RNAs (ceRNAs) in CRC remain unclear. In this study, we constructed an exosomal ceRNA network to identify the core ceRNAs and investigate the diagnostic biomarkers in CRC.

**Methods and Patients:** Serum exosomes were isolated from four CRC patients and two healthy donors by ultracentrifugation, and then subjected to RNA isolation, sequencing and microarray. Kyoto Encyclopedia of Genes and Genomes (KEGG) pathway and Gene Ontology (GO) analyses were performed to identify functional enrichment implications of differentially expressed exosomal mRNAs. TargetScan and miRanda were used for identifying the miRNA-mRNA and miRNA-LncRNA interactions. The predicted lncRNAs and mRNAs were intersected with the differentially expressed genes, for which the screening criterion was fold change >1.5 in the microarray. Differentially expressed exosomal miRNAs were identified in the GSE71008 dataset, and differentially expressed mRNAs (DEmRNAs) were further summarized from The Cancer Genome Atlas (TCGA) database.

**Results:** A total of 1186 exosomal DEmRNAs, 2088 exosomal DElncRNAs and 29 exosomal miRNAs were detected in CRC patients compared to the healthy donors. Functional enrichment analysis suggested that exosomal DEmRNAs might participate in pathways related to carcinogenesis and development of cancer. An exosomal ceRNA regulatory network of CRC was constructed based on 40 lncRNAs, two miRNAs, and five mRNAs. Exosomal miR-150-5p and miR-10b-5p expression levels were increased in healthy donors compared with CRC patients in the GSE71008 dataset, and five DEmRNAs (*TOMM70A*, *RBM48*, *BEND3*, *RHOBTB1*, and *ADAMTS2*) were significantly upregulated in TCGA database. Two potential exosomal regulatory axes of lncRNA G016261-miR-150-5p-*RBM48* and lncRNA XLOC_011677-miR-10b-5p-*BEND3* were identified from the network.

**Conclusion:** The current study revealed potential molecular biological regulation pathways and diagnostic biomarkers through the exosomal ceRNA regulatory network.

## Introduction

More than 1.8 million new cases of colorectal cancer (CRC) and 881,000 deaths were estimated to occur in 2018 [1]. CRC is the fourth most commonly diagnosed malignancy and the third leading cause of cancer-related mortality, which emphasizes the need for screening and early detection of CRC. Recently, non-invasive procedures have been developed for CRC pre-screening, such as liquid biopsy, which underlines new approaches for cancer detection, including circulating tumor nucleic acids (ctDNA and ctRNA), circulating tumor cells (CTCs), exosomes, etc [2].

Exosomes are 50–150 nm microvesicles that play a crucial role in cell–cell communication [3–5]. Through ligand-receptor interactions, endocytosis and phagocytosis, exosomes interact with the method by which plasma fusion transfers information to the adjacent target cell membrane. This exosomal crosstalk involves the transport of proteins, mRNAs, miRNAs and lncRNAs between donor and recipient cells [6, 7], leading to cancer initiation, progression and metastasis as well as epithelial–mesenchymal transition (EMT), angiogenesis, drug resistance and immune escape [8–10]. Many types of exosomal miRNAs, lncRNAs and mRNAs have been identified, and the underlying molecular mechanisms have been investigated, but their potential networks have not been fully elucidated.

The hypothesis of competing endogenous RNAs (ceRNAs) highlights a specific molecular biological regulatory mechanism for post-transcriptional regulation. Serving as ceRNAs, lncRNAs could indirectly regulate the expression of mRNAs through competitive binding response elements of miRNAs as endogenous molecular sponges, thereby regulating gene expression and cell function [11, 12]. Several studies have revealed that ceRNA networks play an important role in regulating tumor cell proliferation, differentiation, metastasis and chemo-resistance [13–15]. LncRNA-FAM225A binds to miR-1275 and miR-590-3p to regulate the expression of *ITGB3*, which is a potential ceRNA regulatory pathway that ultimately promotes tumorigenesis and metastasis in nasopharyngeal carcinoma [16]. LncRNA-KRTAP5-AS1 and lncRNA-TUBB2A could act as ceRNAs to affect the function of Claudin-4 by miR-596 and miR-3620-3p in gastric cancer [17]. Exosomal lncRNA-Sox2ot from Hs 766T-L2 cells transfers to BxPC-3 cells and modulates the expression of Sox2 by competitively binding to the miR-200 family, thus promoting invasion and metastasis of pancreatic ductal adenocarcinoma (PDAC) [18]. Exosomal lncRNA-UCA1 sequesters miR-135a, miR-143, miR-214 and miR-1271 to protect *ANLN*, *BIRC5*, *IP O 7*, *KIF2A*, and *KIF23* from miRNA-induced degradation in CRC [19]. Taken together, these findings suggested that exosomes could transfer RNAs, especially noncoding RNAs, to the recipient cells and participated in ceRNA network interactions to regulate specific aspects of tumor development, thereby indicating that exosomal ceRNA complex can act as excellent biomarkers for CRC diagnostics.

In this study, we first selected the differentially expressed exosomal lncRNAs between CRC patients and healthy donors using microarray and an integrated analysis, and then predicted and verified their combined miRNAs and mRNAs by bioinformatics prediction and correlation analysis using GEO and TCGA databases. Finally, we constructed the ceRNA network interactions in CRC and explored the potential role of exosomes involved in this network.

## Materials and Methods

### Patients and Samples

Four CRC patients and two healthy donors, admitted at the Shandong Cancer Hospital between september 2017 and July 2018, were selected for this study. Written informed consent was obtained from all participants. The clinical stage of the CRC patients was determined, and one patient was classified as stage I, one as stage II, and two patients as stage IV according to the 8 AJCC Cancer Staging Handbook of the American Joint Committee on Cancer. The protocol was approved by the Shandong Cancer Hospital and Institute, Shandong First Medical University and Shandong Academy of Medical Sciences of committee. All subjects gave written informed consent in accordance with the Declaration of Helsinki.

CRC patients were not received any tumor treatment or suffered from any other immune, metabolic diseases or endocrine before serum collection, and all patients had clearly pathologically diagnosed as colorectal cancer, four of which were adenocarcinoma. Healthy donors do not have any underlying diseases, especially gastrointestinal diseases. Four CRC patients and two healthy donors were from 18 to 65 years old.

### Isolation of Exosomes

The exosomes were isolated by ultracentrifugation, as previously described [20]. Briefly, the serum was centrifuged at 10000 ×g for 30 min at 4°C to remove the cellular debris, and then ultracentrifuged at 100,000 ×g (Beckman Coulter, Brea, CA, United States) at 4°C for 2 h to precipitate the exosomes. Trizol was added to the precipitate, and the samples were stored at −80°C.

### Transmission Electron Microscopy

The exosome pellets were placed to the copper grids in a 50 µL drop of 1% glutaraldehyde for 5 min and transferred to a 100-µL drop of distilled water, after 2 min, the grids were stained with to a 50-µL drop of uranyl-oxalate solution (pH 7) for 5 min and placed a parafilm-covered glass dish covered anon ice. Subsequently, the exosome grids were cleaned using distilled water seven times for 2 min each. The TEM images were obtained using a JEM-1200EX transmission electron microscope (JEOL, Japan) operated at 100 kV.

### Tunable Resistive Pulse Sensing

The size of the vesicles and the particle concentration of exosomes were measured by using TRPS (qNano; Izon Science Ltd.). The tunable nanopores with proprietary data capture and analysis using Izon Control Suite software v.3.3.2.2000 (Izon Science Ltd.).

### Western Blotting Analysis

The extracted protein was resolved by 10% SDS-PAGE, transferred onto the PVDF membranes and placed in blocking buffer for 2 h, the membranes were incubated overnight with anti-CD63, anti-GM130, anti-TSG101 (CST, Danvers, United State) at 4°C, followed by incubation with HRP-coupled secondary Ab at room temperature for 1 h. The protein bands were detected through an ECL blot detection reagent.

### Analysis of miRNA Sequence Datasets

The input material for the small RNA library was generated from 3 μg RNA in each sample. After the cluster was generated, the libraries were sequenced and 50 bp single-end reads were generated on the Illumina HiSeq 2500/2000 platform (Illumina, United States). After sequencing, coding potential analysis, quality control analysis, gene expression level quantification, transcriptome assembly, conservative analysis, target gene prediction, read mapping to the reference genome and differential expression analysis were carried out by Novogene Corporation.

### Differential mRNA and LncRNA Expression of RNA-Microarray Datasets and Analysis

Global analysis of human lncRNA and protein-coding transcripts was conducted using human lncRNA microarray V4.0. Briefly, after the rRNA was removed using the mRNA-ONLY™ eukaryotic mRNA isolation kit (Epicentre), the mRNA was purified from the total RNA. Then, a random primer method (Arraystar flash RNA labeling kit) was used to amplify each sample and transcribe it into fluorescent cRNA along the entire length of the transcript without 3’bias. RNeasy mini kit (Qiagen) was used to purify the labeled cRNAs. The concentration and specific activity of the labeled cRNAs were measured by NanoDrop ND-1000. The labeled cRNA was diluted using 50 μl of hybridization solution, distributed on the spacer slides, and placed on the lncRNA expression microarray slides. The slides were incubated at 65°C for 17 h in an Agilent hybridization oven. After washing, the slides were fixed and scanned using Agilent DNA Microarray Scanner (Part No. G2505C).

### Exosomal ceRNA Network Construction

To identify potential pairs among exosomal DEmRNAs, DElncRNAs, and DEmiRNAs between the CRC patients and healthy donors, data from two databases were retrieved. Arraystar’s in-house miRNA target prediction software was applied based on TargetScan and miRanda algorithms. We intersected the predicted targets obtained from the database with miRNA sequencing and lncRNA, mRNA microarray. Fold-change of >1.5 and a *p*-value of <0.05 were set as the criteria.

Thereafter, the miRanda and TargetScan databases were used to predict potential miRNA targets, and Cytoscape software was used to delineate the ceRNA network of lncRNA-miRNA-mRNA.

### Functional Annotation of the Exosomal ceRNA Network

The Gene Ontology (GO) project describes the attributes of genes and gene products in any organism (http://www.geneontology.org). The ontology includes three domains: Cellular Component, Molecular Function and Biological Process. The *p*-value indicates the importance of GO enrichment in the DE gene. The lower the *p*-value, the more significant is the GO term (*p*-value ≤0.05). The functional analysis of gene mapping to KEGG pathway is called pathway analysis. The *p*-value indicates the importance of disease-related pathways. Lower the *p*-value, more significant is the pathway. The recommended *p*-value cutoff is 0.05.

### Statistical Analysis

GraphPad Prism 6.0 (San Diego, CA, United States) and SPSS 22.0 (IBM, Ehningen, Germany) were used for the statistical analyses. Mann–Whitney U or *t*-test were used to perform the comparisons. Receiver operating characteristic (ROC) curves with area under the curve (AUC) were used to determine the corresponding cutoff values, with pathological diagnosis as the “gold standard”. A *p*-value <0.05 was considered to be statistically significant. In addition, each combination of miRNA and mRNA or mRNA was analyzed.

## Results

### Identification of Isolated Exosomes

**FIGURE 1:**
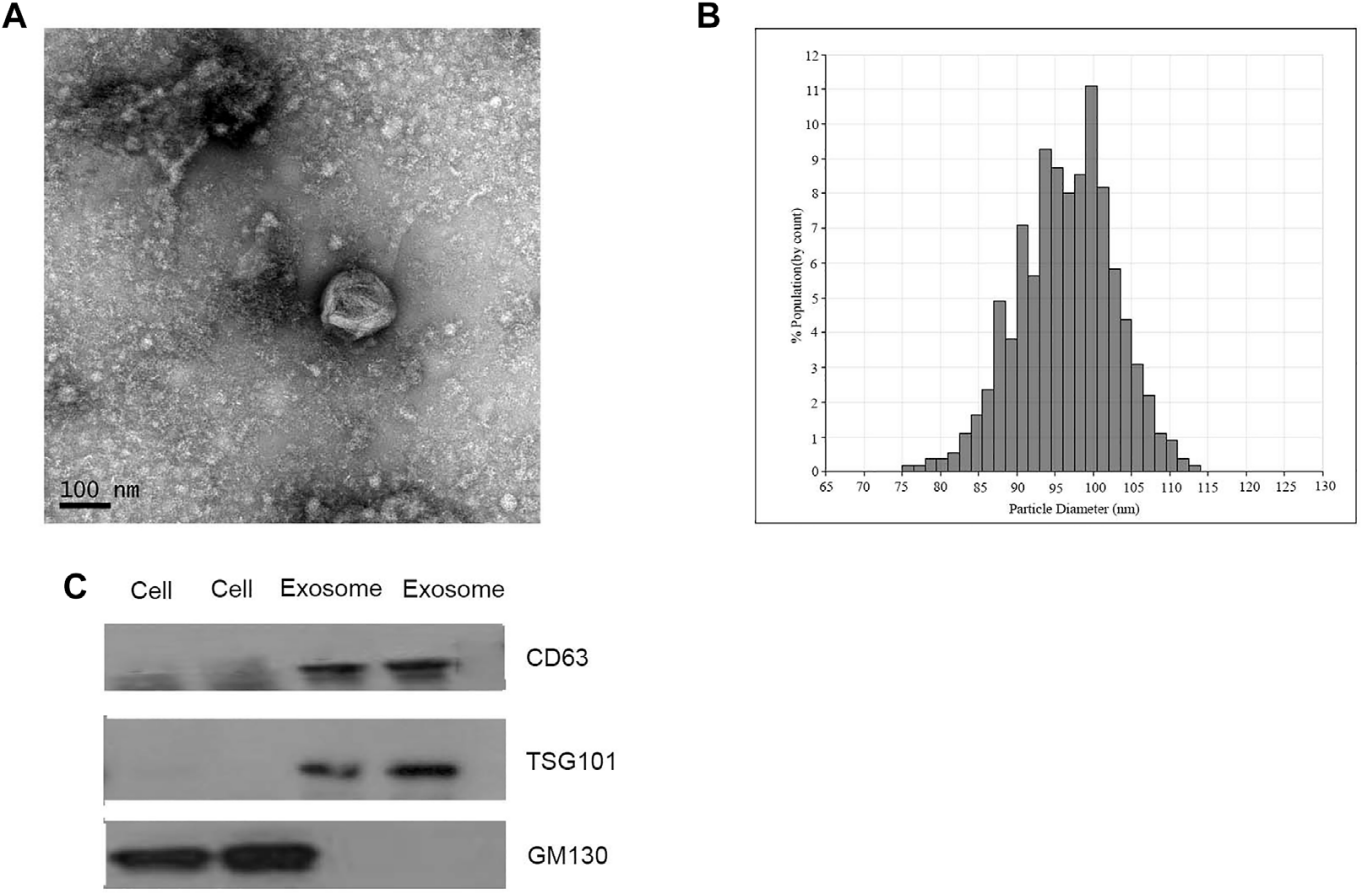
Identification of isolated exosomes. **(A)** TEM image of exosomes from CRC patients with 50–150 nm diameter (scale bar: 100 nm; high voltage (HV) = 100 kV). **(B)** Size distribution of exosomes with 50–150 nm diameter were measured by qNano system. **(C)** Western blot analysis of CD63, TSG101, and GM130 as exosomal markers.

Exosomes isolated from CRC patients and healthy donors were characterized by qNano, TNM and western blotting analysis. The results suggested the typical exosome-like round morphology with 50–150 nm in diameter under TEM (Figure 1A), and the size range was in agreement with qNano (Figure 1B). Consistently, the characteristic protein markers of exosomes, CD63 and TSG101 were found in exosomes, but not in the whole cell lysates. whereas GM130 (the negative control) was not detected in the exosomal protein lysates (Figure 1C).

### Identification of Exosomal DE mRNAs, DE lncRNAs, and DE miRNAs

**FIGURE 2:**
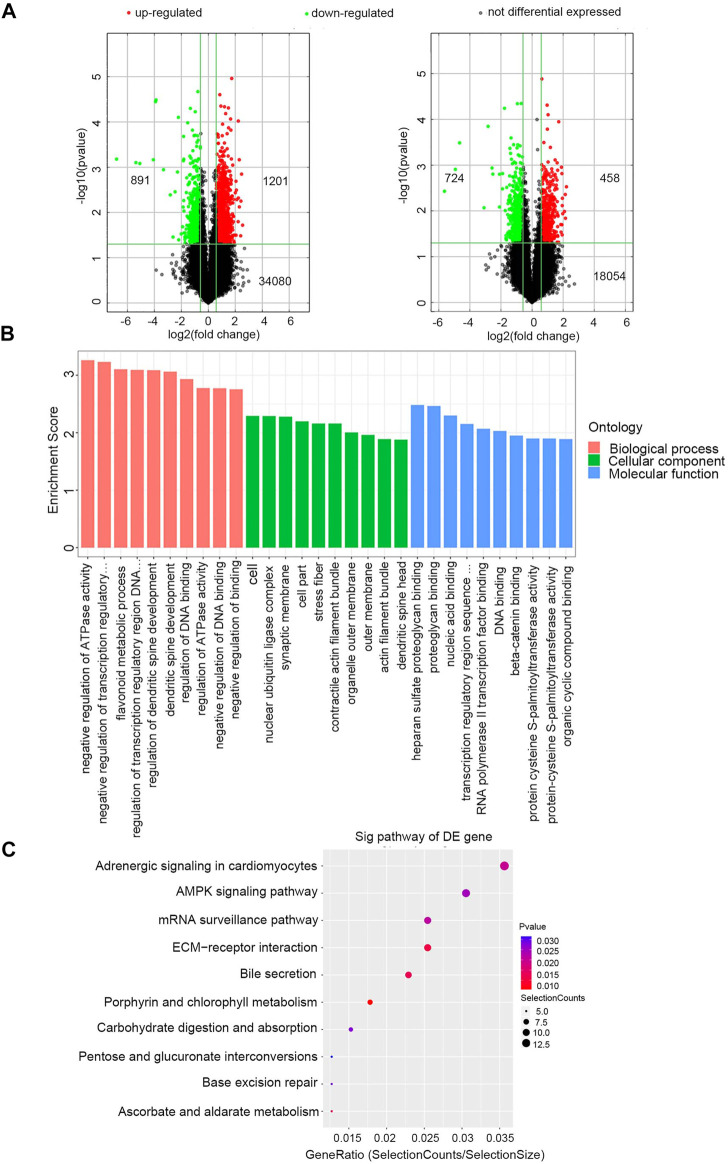
Identification of exosomal DERNAs and Gene ontology and pathway analysis of DEmRNAs **(A)** Exosomal lncRNA and mRNA were compared by the volcano graph the expression fold changes in healthy donors and CRC patients. The green dots indicated the downregulated lncRNAs and mRNAs, the red dots indicated the upregulated lncRNAs and mRNAs and the black dots represent no differential expressed. **(B)** DE mRNAs were clustered in top 10 terms from a GO analysis of molecular function, biological process and cellular component. **(C)** DE mRNAs were clustered in Kyoto Encyclopedia ofGenes and Genomes (KEGG) pathways.

Exosomal RNAs from four CRC patients and two healthy donors were isolated for RNA sequencing and microarray. A total of 28189 downregulated genes (10808 mRNAs, 16840 lncRNAs, and 541 miRNAs) and 28364 upregulated genes (8428 mRNAs, 19332 lncRNAs, and 604 miRNAs) were screened in CRC patients compared to healthy donors, of which 1182 DE mRNAs and 2092 DE lncRNAs were selected with the thresholds of *p*-value <0.05 and fold-change >1.5 (Figure 2A). Exosomal miRNAs have been reported in our previous study [21].

The GO and KEGG enrichment analyses of these 1182 exosomal DE mRNAs were further conducted to investigate the possible signaling mechanisms of CRC. Interestingly, exosomal DE mRNAs were mainly enriched in negative regulation of ATPase activity in the biological process classification, and nucleic acid binding in the molecular function classification (Figure 2B). Additionally, KEGG pathway analysis showed that 207 pathways were significantly enriched. The top 10 significantly enriched pathways are shown in Figure 2C, including ECM-receptor interaction, adenosine monophosphate-activated protein kinase (AMPK) signaling pathway and mRNA surveillance pathway, which are closely correlated with the carcinogenesis and development of CRC. Therefore, these findings provided a resource for additional molecular participants in CRC.

### Expression Analysis of Exosomal DE lncRNA-DE miRNA Pairs in Colorectal Cancer

**FIGURE 3:**
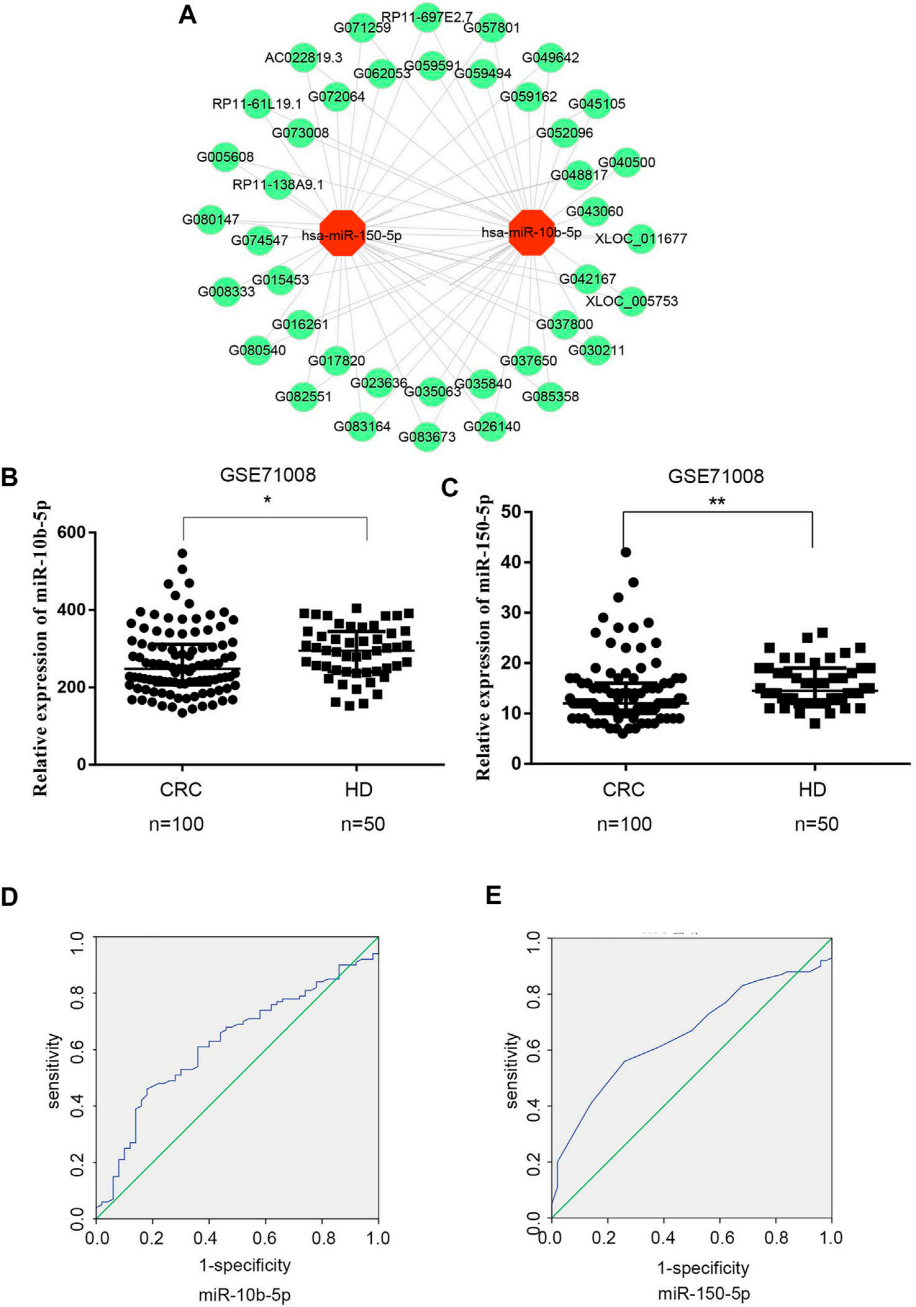
Expression analysis of exosomal DElncRNA-DEmiRNAs pairs in CRC **(A)** Exosomal DElncRNA-DEmiRNAs pairs, the green dots represents up-regulated DElncRNAs, the red dots represents DEmiRNAs **(B)** Mann‐Whitney U test shows that there is a significant differences in miR-10b-5p expression levels between CRC patients and healthy donors in GSE71008 dataset. Data were expressed as median with interquartile range (*p = 0.0125). **(C)** Mann‐Whitney U test showed that there is a significant difference in miR-150-5p expression levels between CRC patients vs. healthy donors in GSE71008 dataset. Data were expressed as median with interquartile range (*p* = **0.0014). **(D,E)** The AUC of exosomal miR-10b-5p and exosomal miR-105-5p was 0.625 and 0.658 in 100 CRC patients vs. 50 healthy donors.

To explore the relationship between exosomal DE lncRNAs and DE miRNAs in CRC, we predicted the target miRNAs of top 40 DE lncRNAs in the microarray expression profiles using TargetScan and miRanda databases. Finally, miRNA-150-5p and miRNA-10b-5p were selected since they could be targeted by the majority of exosomal DE lncRNAs (Figure 3A).

The two exosomal DE miRNAs were also analyzed using the GEO database, which includes exosomal RNA-sequence profiles from 100 CRC patients and 50 healthy donors (GSE71008). In the database, the exosomal miR-150-5p and miR-10b-5p expression levels were markedly downregulated in CRC patients (*p* < 0.0125 and *p* < 0.0014, respectively) compared to healthy donors (Figures 3B,C). In order to analyze the diagnostic performance of exosomal miR-150-5p and miR-10b-5p for CRC, a ROC curve was calculated. The AUC of miR-10b-5p was 0.625 (95% CI: 0.567–0.689), with specificity of 82% and sensitivity of 46%, while the AUC of miR-150-5p was 0.658 (95% CI: 0.571–0.745), with sensitivity of 56% and specificity of 58.8% (Figures 3D,E). Taken together, these data suggested that exosomal miR-150-5p and miR-10b-5p were significantly associated with CRC.

### Expression Analysis of Exosomal DE miRNA-DE mRNA Interaction in Colorectal Cancer

**FIGURE 4:**
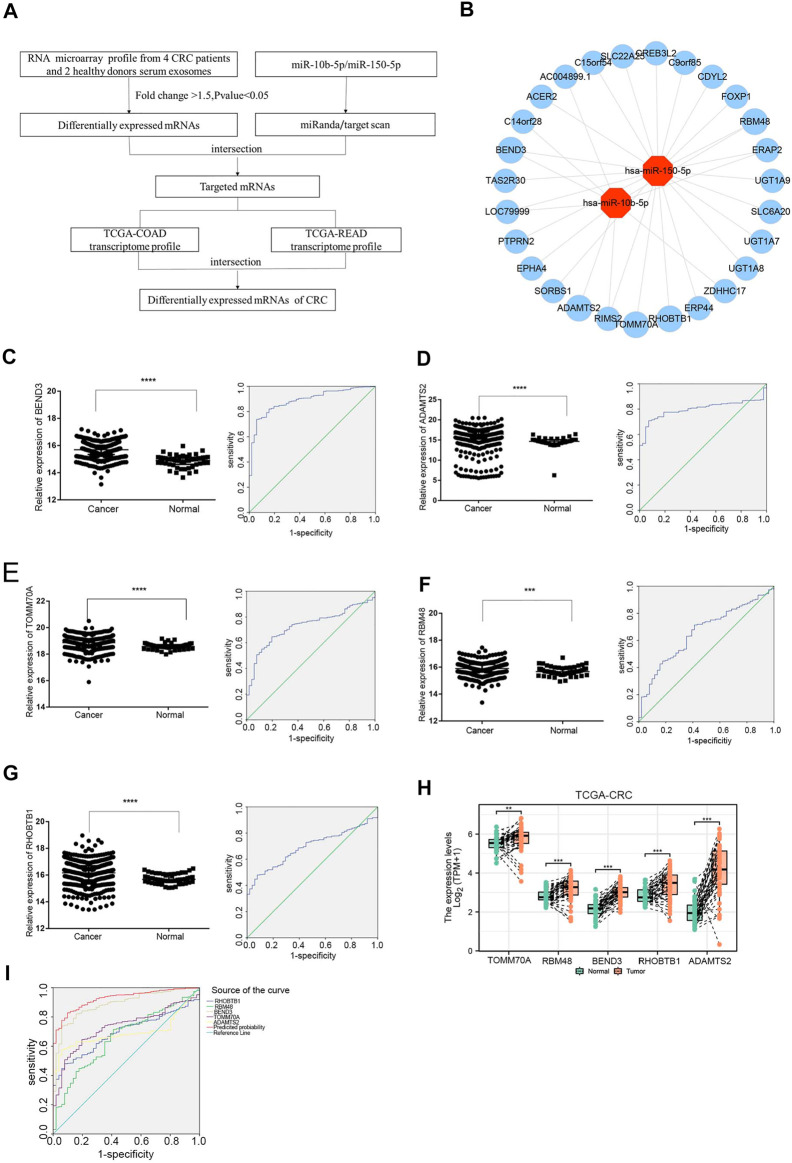
Expression analysis of exosomal miRNA-mRNA internections in CRC **(A)** Flowchart of the DEmRNAs in CRC. TCGA-COAD: The Cancer Genome Atlas-colon adenocarcinoma, TCGA-READ: The Cancer Genome Atlas-rectal adenocarcinoma. **(B)** Analysis of exosomal miR-10b-5p and miR-150-5p targeted up-regulated DEmRNAs, red dots represent DEmiRNAs and the blue dots reprsent DEmRNAs. **(C–G)** The relative expression of *BEND3*, *ADAMTS2*, *TOMM70A*, *RBM48*, *RHOBTB1* from TCGA database (CRC patients *n* = 623, Healthy donors *n* = 51). The AUC of *BEND3*, *ADAMTS2*, *TOMM70A*, *RBM48*, *RHOBTB1* were 0.887, 0.695, 0.73, 0.66, 0.692 for CRC diagnostics, respectively. **(H)** *BEND3, ADAMTS2, TOMM70A, RBM48 and RHOBTB1* were significantly up-regulated in CRC tissues compared to the precancerous in the TCGA database. **(I)** The combination of five DEmRNAs had an AUC of 0.925 with a specificity of 90.2% and sensitivity of 82.8%.

The study flowchart was shown in Figure 4A. To identify targeted DE mRNA pairs, we predicted targeted mRNAs from the TargetScan and miRanda databases, followed by intersecting the predicted mRNAs with the exosomal DE mRNAs in the microarray, and finally constructed 27 mRNAs targeted by miR-10b-5p and miR-150-5p in the CRC regulatory network (Figure 4B). Due to lower complexity and more reliability, five significantly upregulated mRNAs (*TOMM70A*, *RBM48*, *BEND3*, *RHOBTB1* and *ADAMTS2*) in CRC patients (*n* = 623) compared to healthy donors (*n* = 51) were identified and analyzed using data from TCGA. As shown in Figures 4C–G, ROC curves were also calculated, and the AUCs of *BEND3*, *ADAMTS2*, *TOMM70A*, *RBM48* and *RHOBTB1* were 0.887, 0.695, 0.73, 0.66 and 0.692, respectively. The study found that *BEND3*, *ADAMTS2*, *TOMM70A*, *RBM48* and *RHOBTB1* were significantly up-regulated in CRC tissues compared to the precancerous in the TCGA database (Figure 4H). Notably, the AUC of the combination of these five mRNAs was 0.925 (95% CI: 0.899-0.951), with specificity and sensitivity of 90.2% and 82.8%, respectively (Figure 4I).

### Construction and Correlation Analyses of the Exosomal ceRNA Network

**FIGURE 5:**
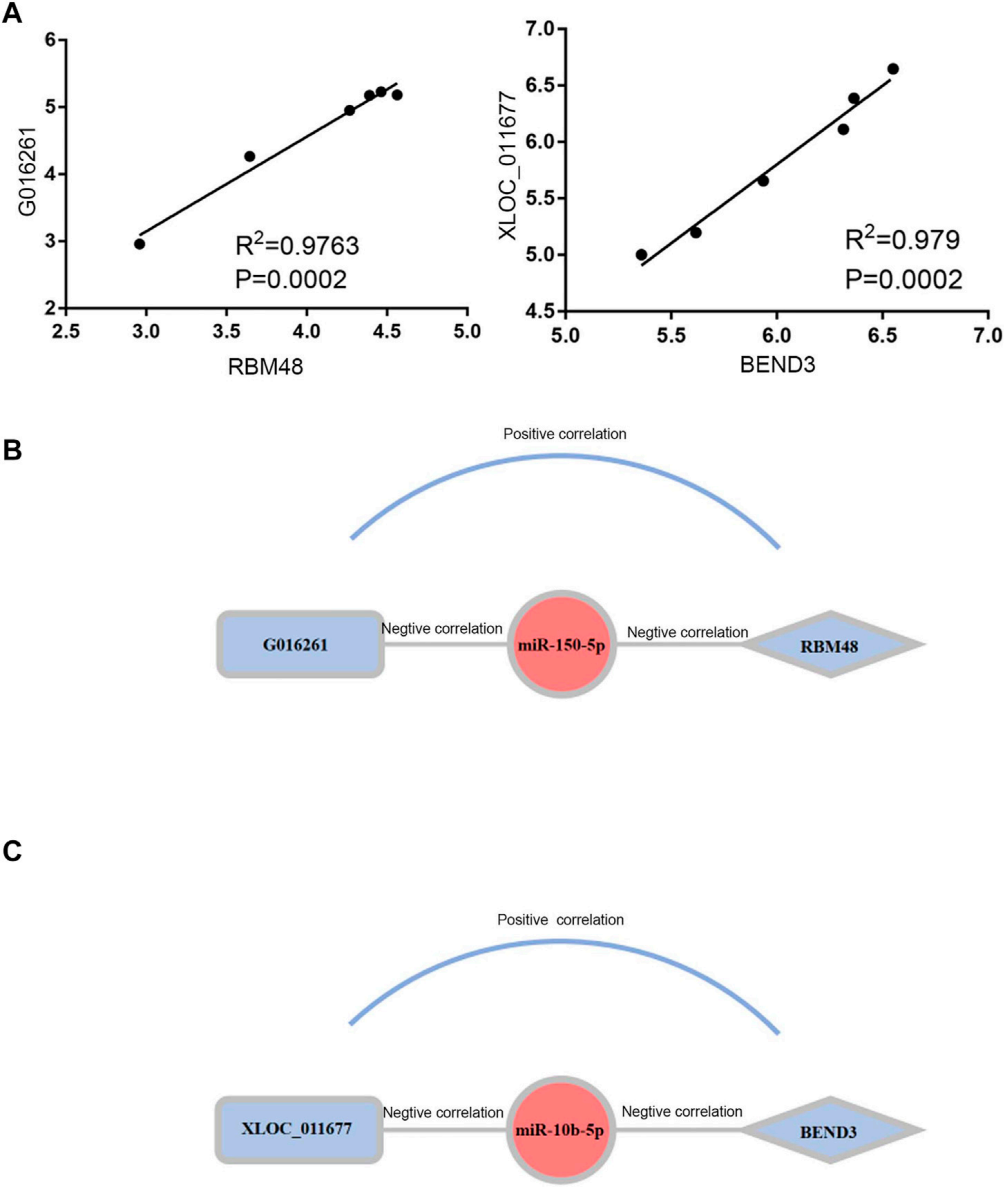
Construction and Correlation analyses of exosomal ceRNA network in CRC. **(A)** Pearson correlation analysis for exosomal DElncRNAs and associated DEmRNAs. Horizontal axis indicates normalized exosomal DElncRNA expression levels; vertical axis indicates normalized exosomal DEmRNA expression levels (lncRNA xloc_011677 correlates to *BEND3*, lncRNA G016261 correlates to RBM48).**(B,C)**The exosomal lncRNA G016261-miR-150-5p-*RBM48* and exosomal lncRNA XLOC_01167-miR-10b-5p-*BEND3* regulatory axis, miRNAs, lncRNAs and mRNAs are represented by ellipse, round rectangle and diamonds, respectively.

To elucidate the regulatory mechanism of CRC, an exosomal lncRNA-related ceRNA network of CRC was developed based on the above results. The ceRNA hypothesis states that exosomal lncRNAs harboring MREs can competitively bind to certain miRNAs, thus regulating miRNA-mediated downstream target gene silencing at the post-transcriptional level. We found that 40 exosomal DE lncRNAs might competitively bind to miR-10b-5p and miR-150-5p, and the latter might regulate 27 downstream targeted mRNAs (Figure 4B), which explained how mRNA expression was regulated by exosomal lncRNAs by combining with miRNAs. To confirm the possibility of indirect interactions between DE lncRNAs and DE mRNAs, a Pearson’s correlation analysis was performed with R2 ≥0.95 and *p* < 0.001 as criteria, which revealed a positive correlation between the expression levels of ten DE lncRNA-DE mRNA pairs (Figure 5A). Among which exosomal lncRNA G016261/*RBM48* and lncRNA XLOC_011677/*BEND3* were better matched than others in binding sites, thus lncRNA G016261/*RBM48* and lncRNA XLOC_011677/*BEND3* were selected (Tables 1, 2).

**TABLE 1:** The targets mRNAs and lncRNAs of the miR-150-5p in targetsan and miRanda.

GeneSymbol	Type	8mer	7mer-m8	7mer-A1	6mer	Offset 6mer	Imperfect
*RBM48*	mRNA	1	0	0	0	0	1
*TOMM70A*	mRNA	0	1	0	0	0	0
*BEND3*	mRNA	0	1	0	0	0	0
G045105	lncRNA	0	1	0	0	0	0
XLOC_011677	lncRNA	0	2	0	0	0	0
G074547	lncRNA	0	1	0	0	0	0
G083164	lncRNA	0	1	0	0	0	0
*RHOBTB1*	mRNA	0	2	0	0	0	0
G016261	lncRNA	0	1	0	0	0	0
G059494	lncRNA	0	0	0	0	0	1
G005608	lncRNA	0	0	0	0	0	0

**TABLE 2:** The targets mRNAs and lncRNAs of the miR-10b-5p in targetsan and miRanda.

GeneSymbol	Type	8mer	7mer-m8	7mer-A1	6mer	Offset 6mer	Imperfect
G045105	lncRNA	0	1	0	0	0	0
XLOC_011677	lncRNA	1	1	0	0	0	0
G016261	lncRNA	1	2	0	0	0	0
G059591	lncRNA	0	1	0	0	0	0
ADAMTS2	mRNA	0	1	0	0	0	0
G083164	lncRNA	0	1	0	0	0	0
BEND3	mRNA	1	0	0	0	1	0
RBM48	mRNA	0	1	0	0	0	0
G059494	lncRNA	0	1	0	0	1	0
G005608	lncRNA	0	0	0	0	0	0

8mer: 2-8 nucleic acid perfect matching, and the first nucleic acid was A. 7mer-m8: 2-8 nucleic acid perfect matching, and the first nucleic acid wasn’t A. 7mer-A1: 2-7 nucleic acid perfect matching, and the first nucleic acid was A. 6mer: 2-7 nucleic acid perfect matching, and the first nucleic acid was A. Offset 6mer: 3-8 nucleic acid perfect matching. Imperfect: 2-7 nucleic acid was mismatch or deleted.

Finally, we predicted the exosomal ceRNA network in CRC. As shown in Figure 5B, lncRNA G016261 would function as ceRNA that interact with *RBM48*, serving as miRNA sponges to restrain miR-150-5p function, then inhibit its regulation of target gene *RBM48* expression; Meanwhile, lncRNA XLOC_011677 may also act as ceRNA to compete with *BEND3* transcripts for the miR-150-5p, thus mediating the interaction and regulation between miR-10b-5p and *BEND3* (Figure 5C).

## Discussion

Globally, colorectal cancer (CRC) is the third most commonly diagnosed malignancy and the second leading cause of cancer-related deaths [22]. Exosomes function as potent cell-cell transfer cargos for intercellular transfer of bioactive molecules, including lncRNA to the recipient cells and participate in ceRNA network. Recently, ceRNA hypothesis has been proposed to represent a class of RNAs with miRNA binding sites that competitively bind to miRNAs to inhibit their regulation of target genes, playing a crucial role in the underlying molecular mechanisms of CRC initiation and progression.

To verify the effectiveness and practicality of exosomal miRNAs, we compared them with another dataset, GSE71008, which includes exosomal RNA-sequence profiles from 100 CRC patients and 50 healthy donors. GSE71008 showed that compared with healthy donors, the expression of exosomal miR-150-5p and miR-10b-5p were markedly decreased in CRC patients. Previous studies reported that miR-10b-5p and miR-150-5p were associated with cancer progression, proliferation, and migration [23–25]. MiR-150-5p was significantly downregulated in both plasma and cancer tissue samples, and might be a biomarker in cancer [21, 26, 27]. Chen et al. showed that miR‐150‐5p inhibited CRC cell proliferation, migration, invasion and angiogenesis *in vitro* and *in vivo*, and its inhibitory effect could be reversed by vascular epithelial growth factor A (*VEGFA*) [28]. Other studies revealed a significantly lower expression of miR-10b-5p in renal cell carcinoma and triple-negative breast cancer [25, 29]. Jin et al. reported that exosomal miR-10b-5p may be a promising and effective candidate for the development of highly sensitive, non-invasive biomarkers for early NSCLC diagnosis [30]. MiR-10b-5p is related to proliferation, migration, and invasion of CRC cells [31].

MiRNA regulates gene expression at the post-transcriptional level by targeting mRNA 3′UTR. Based on microarray data and TCGA database analysis, we identified miR-10b-5p and miR-150-5p target mRNA pairs, which included *TOMM70A*, *RBM48*, *BEND3*, *RHOBTB1* and *ADAMTS2*. The five mRNAs were markedly upregulated in CRC patients compared with healthy donors from TCGA database. Bai et al. identified that RBM48 was a U12 splicing factor that promoted cell differentiation and repressed cell proliferation, and U12 was associated with the occurrence and development of cancer. BEND3 is a nuclear protein and transcriptional repressor that associates with the HP1-containing heterochromatin loci [32, 33], and might play a role in identifying the subgroup of breast carcinoma patients with potential to develop visceral metastasis [34]. *TOMM70A* localizes in the mitochondria of COS-7 cells [35], and is a new biomarker of resistance to hormonal therapy in breast cancer [36, 37]. *RHOBTB1* is an atypical Rho GTPase with two BTB domains in addition to its Rho domain. Chemotherapy-induced fatigue may involve downregulation of *RHOBTB1* in peripheral blood mononuclear cells of patients with locoregional breast cancer [38]. Many studies have shown that tumor suppressor *RHOBTB1* contributes to the proliferation and invasion of cancer [39–41]. *ADAMTS*-2 is associated with tumor progression and invasion, metastasis and CRC-specific survival, and may serve as a potential biomarker to stratify CRC patients into low and high risk of tumor metastasis [42]. Jiang et al. reported that *ADAMTS*-2 protein expression was remarkably higher in gastric cancer cells than in normal tissues, therefore, *ADAMTS*-2 may be a potential biomarker for assessing the prognosis of gastric carcinoma [43].

In summary, a ceRNA regulatory network was successfully developed by identification of cancer‐specific lncRNAs, miRNAs and mRNAs. We propose that this regulatory network centered on exosomal miR-10b-5p and miR-150-5p may play a critical role in the carcinogenesis of CRC. This study highlighted a novel ceRNA mechanism in which exosomal lncRNA G016261 and lncRNA XLOC_011677-sponging miR-10b-5p and miR-150-5p regulate the expression of *RBM48* and *BEND3*. A diagnostic model based on miRNAs in the ceRNA network might help in improving the diagnosis efficiency for CRC patients compared to miRNAs alone. However, exosomal lncRNA G016261-miR-150-5p-*RBM48* and lncRNA XLOC_011677-miR-10b-5p-*BEND3* regulatory axes should be further studied to fully elucidate their biological functions and confirm the molecular mechanisms.

## Data Availability

The original contributions presented in the study are included in the article/Supplementary Material, further inquiries can be directed to the corresponding author.
