# Supplementary material for: Identification of Diagnostic Exosomal LncRNA-miRNA-mRNA Biomarkers in Colorectal Cancer Based on the ceRNA Network
Source: Pathol Oncol Res. 2022 Sep 16;28:1610493. doi: 10.3389/pore.2022.1610493 (PMC9522904; doi:10.3389/pore.2022.1610493)
Supplement: Supplementary file 1 [file DataSheet1.docx]

**Supplementary table 1: Characteristic of sample**

| **Sample ID** | **Histological type** | **Stage type** | **Gender** | **Age(year)** | **Distant metastasis** |
| --- | --- | --- | --- | --- | --- |
| 1 | - | - | Female | 47 | - |
| 2 | - | - | Male | 42 | - |
| 3 | Adenocarcinoma | Ⅰ | Male | 57 | No |
| 4 | Adenocarcinoma | Ⅱ | Female | 61 | No |
| 5 | Adenocarcinoma | Ⅳ | Female | 68 | Yes |
| 6 | Adenocarcinoma | Ⅳ | Male | 55 | Yes |

**1 and 2 were Healthy donor, 3, 4, 5 and 6 were CRC**

**Supplementary table 2: RNA Quantification and Quality Assurance by NanoDrop ND-1000**

| **Sample ID** | **OD260/280 Ratio** | **Volume(μl)** | **Quantity (ng)** | **QC result Pass or Fail** |
| --- | --- | --- | --- | --- |
| 1 | 1.57 | 15 | 742.80 | pass |
| 2 | 1.56 | 15 | 746.10 | pass |
| 3 | 1.59 | 15 | 729.75 | pass |
| 4 | 1.60 | 15 | 742.95 | pass |
| 5 | 1.64 | 15 | 878.40 | pass |
| 6 | 1.60 | 15 | 839.25 | pass |

**1 and 2 were Healthy donor, 3, 4, 5 and 6 were CRC**
